# Stereotactic prostate adaptive radiotherapy utilising kilovoltage intrafraction monitoring: the TROG 15.01 SPARK trial

**DOI:** 10.1186/s12885-017-3164-1

**Published:** 2017-03-08

**Authors:** Paul Keall, Doan Trang Nguyen, Ricky O’Brien, Jeremy Booth, Peter Greer, Per Poulsen, Val Gebski, Andrew Kneebone, Jarad Martin

**Affiliations:** 1 Radiation Physics Laboratory, Sydney Medical School, The University of Sydney, Sydney, NSW Australia; 2 Department of Radiation Oncology, Northern Sydney Cancer Centre, Sydney, NSW Australia; 3 Department of Radiation Oncology, Calvary Mater Newcastle, Newcastle, NSW Australia; 4 Department of Oncology, Aarhus University Hospital, Aarhus, Denmark; 5 University of Sydney NHMRC Clinical Trials Centre, Sydney, NSW Australia

**Keywords:** Stereotactic Radiotherapy, Prostate Cancer, Kilovoltage Intrafraction Monitoring, SPARK Trial

## Abstract

**Background:**

This paper describes the multi-institutional prospective phase II clinical trial, **SPARK**: **S**tereotactic **P**rostate **A**daptive **R**adiotherapy utilizing **K**ilovoltage Intrafraction Monitoring (KIM). KIM is a real-time image guided radiotherapy technology being developed and clinically pioneered for prostate cancer treatment in Australia. It has potential for widespread use for target radiotherapy treatment of cancers of the pelvis, thorax and abdomen.

**Methods:**

In the SPARK trial we will measure the cancer targeting accuracy and patient outcomes for 48 prostate cancer patients who will be treated in five treatment sessions as opposed to the conventional 40 sessions. The reduced number of treatment sessions is enabled by the KIM’s increased cancer targeting accuracy.

**Discussion:**

Real-time imaging in radiotherapy has the potential to decrease the time taken during cancer treatment and reduce the imaging dose required. With the imaging being acquired during the treatment, and the analysis being automated, there is potential for improved throughput. The SPARK trial will be conducted under the auspices of the Trans-Tasman Radiation Oncology Group (TROG).

**Trial registration:**

This trial was registered on ClinicalTrials.gov on 09 March 2015. The identifier is: NCT02397317

## Background

Prostate cancer stereotactic body radiation therapy (SBRT) is becoming one of radiotherapy’s success stories. Technological advances such as the Cyberknife and Calypso have enabled the safe and accurate delivery of high radiation doses to the prostate cancer of eligible patients with high cure rates and low toxicity achieved in five treatment sessions as opposed to the typical 40. Although it is going to take several years for randomized trials comparing SBRT with conventionally fractionated treatments to report, there is already a high adoption of this technology internationally: a U.S. survey showed 64% of centres were using SBRT in 2010, with the SBRT uptake growing at ~10% per year, indicating that by now almost all U.S. centres will be utilising SBRT [1]. The clinical outcome data for prostate cancer SBRT are encouraging and maturing: the recent results of a pooled analysis of 1100 patients demonstrated excellent clinical efficacy, with biochemical disease control rates of greater than 93% at five years, as well as low patient reported toxicity [2, 3]. These excellent efficacy and toxicity profiles have led to ASTRO stating that *‘data supporting the use of SBRT for prostate cancer have matured to a point where SBRT could be considered an appropriate alternative for select patients’* [4].

There is a history of advances in radiotherapy technology improving cancer treatment outcomes. These advances are particularly evident for prostate cancer where both image guided and intensity modulated radiotherapy (IMRT) have independently demonstrated improved tumour control and lower rates of late rectal toxicity [5–8]. However, prostate motion during cancer radiotherapy may shift the tumour outside the beam, simultaneously reducing target dose and exposing normal tissues to potentially damaging radiation doses. The deleterious effects of motion for prostate cancer has led ASTRO to state that ‘*A precise ability to localize the target tumour is essential to fully benefit from SBRT techniques’* [9].

There are several solutions to account for the deleterious effects of motion during prostate cancer radiotherapy, for example the CyberKnife [10], Calypso [11, 12], RayPilot [13] and Real-Time Radiotherapy [14] systems. However, these systems use hardware that is additional to a conventional linear accelerator. A new real-time image guided radiotherapy system, Kilovoltage Intrafraction Monitoring (KIM), uses the x-ray imaging system mounted on the linear accelerator to determine the 3D position of the prostate markers, and by inference the prostate, during radiotherapy treatment. KIM has evolved through *in silico* studies [15, 16], experimental studies [17, 18], the development of a quality assurance program [19], retrospective clinical evaluations [20] and recently clinical deployment in a single institution study (NCT01742403) [21, 22] where the prostate is repositioned if the KIM-guidance system shows motion exceeding a certain threshold, typically 3 mm for 5 s for conventionally fractionated treatments and 2 mm for 5 s for SBRT treatments. The clinical success of KIM has driven the creation of the SPARK trial (NCT02397317) in which KIM will be tested in multiple institutions. This paper describes the SPARK clinical trial.

## Methods/Design

**Fig. 1:**
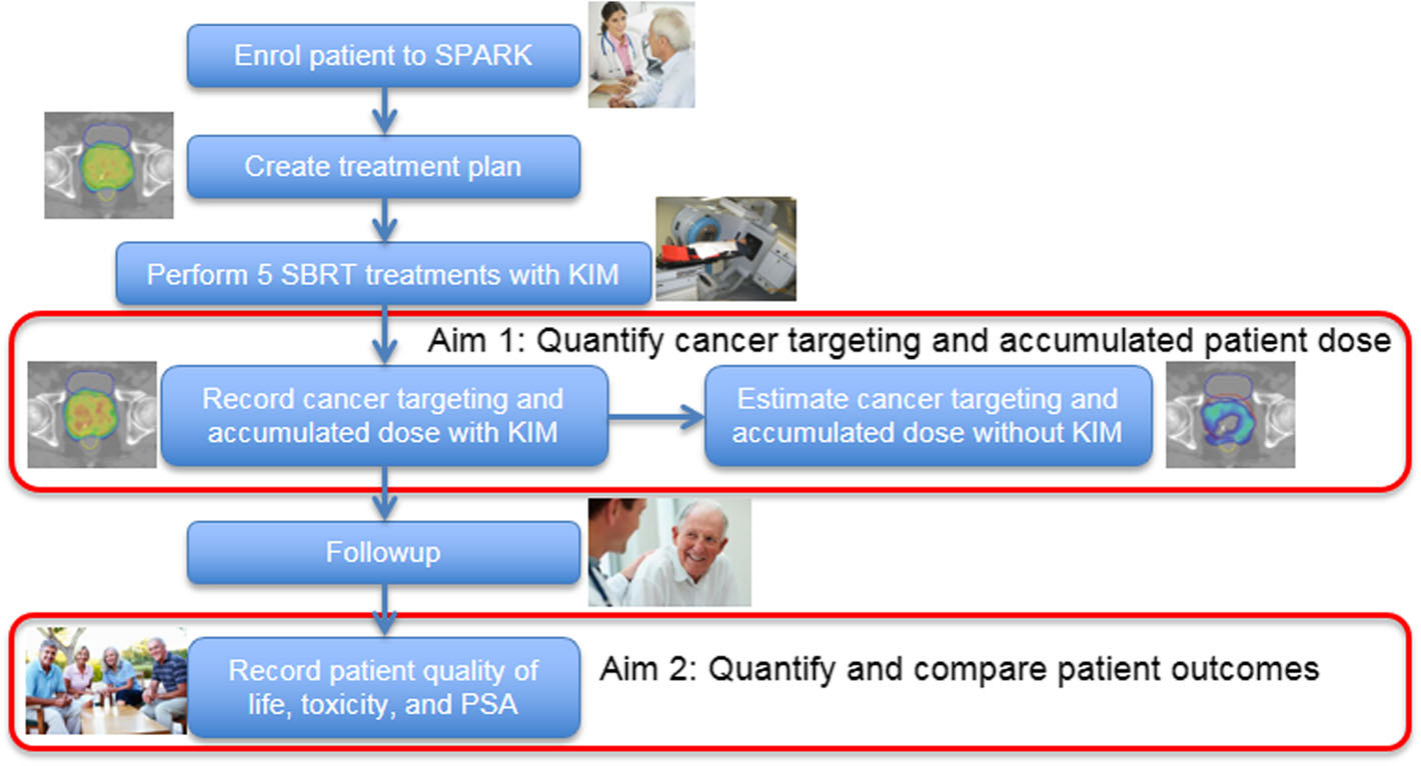
The SPARK study schema

The hypotheses to be tested are that in a phase II clinical trial Stereotactic Prostate Adaptive Radiotherapy utilising KIM (the SPARK trial) improves (1) Patient dose distributions, (2) Patient treatment outcomes and (3) Cancer targeting accuracy. We will test these hypotheses by performing a 48-patient clinical trial at five sites in Australia. The study schema is shown in Fig. 1.

**Fig. 2:**
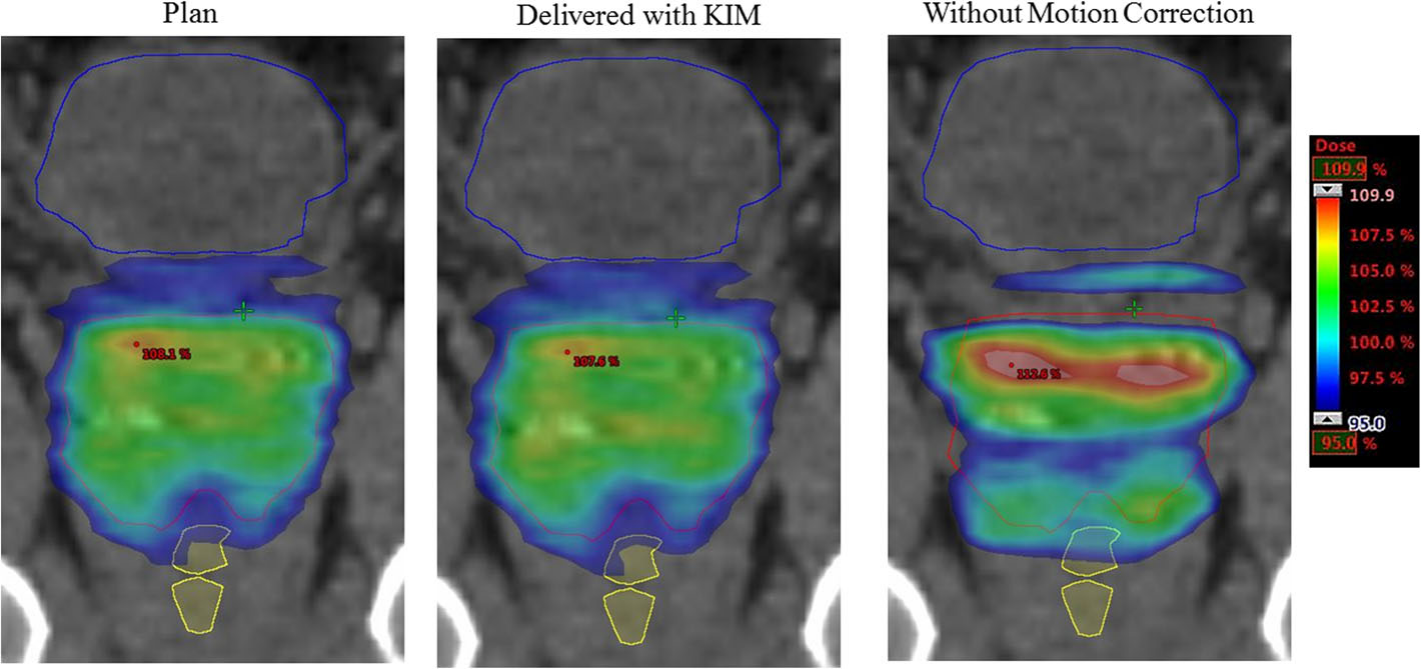
The accumulated patient dose distributions will be quantified via paired control by comparing the accumulated dose distribution from the dose planned (left) with that from KIM corrections (middle) to that which would have been delivered without KIM (right)

**Fig. 3:**
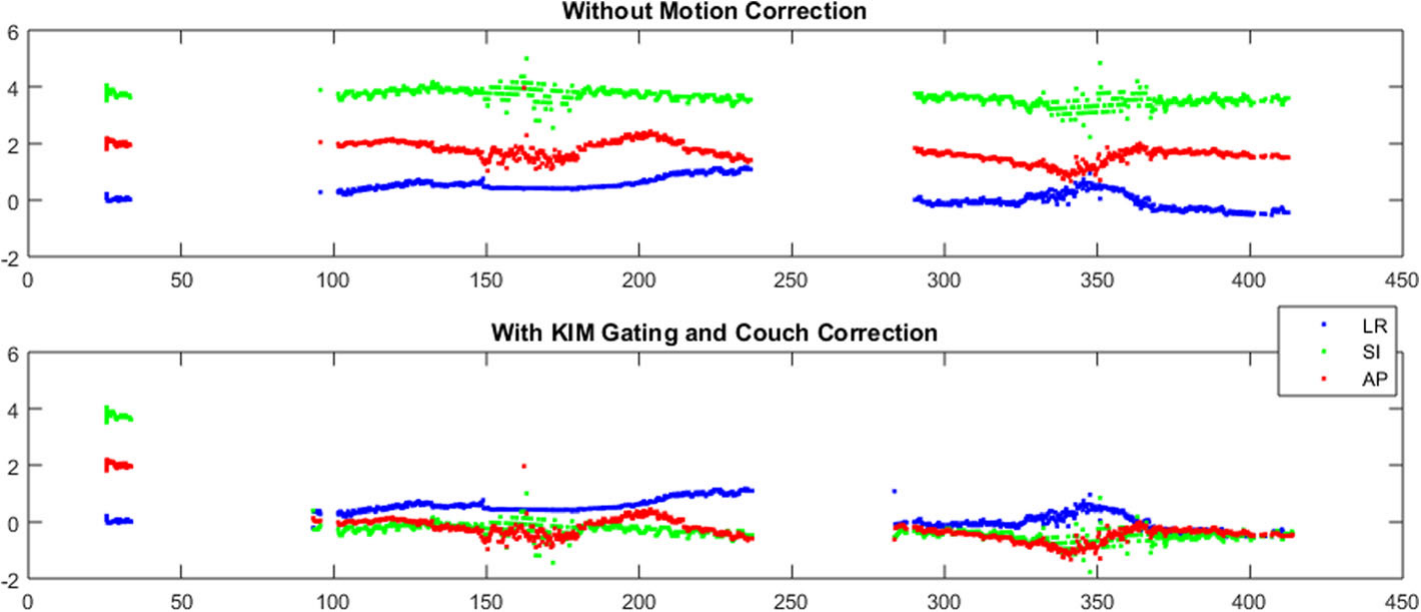
The cancer targeting accuracy will be quantified via paired control by comparing the targeting error that would have been present without KIM (above) to the targeting error with KIM corrections (below)

For each treatment session for each patient, the accumulated patient dose distributions (Fig. 2) and the targeting accuracy (Fig. 3) will be determined via paired control by comparing the measured dose and targeting error with KIM to those that would have been present in the absence of KIM.

### Key selection criteria

#### Inclusion

Aged 18 years or older, histologically proven prostate adenocarcinoma, low or intermediate risk disease as defined by the NCCN guidelines: [23] a) Low Risk: All of PSA < 10 ng/mL, Gleason Grade 6 AND Stage T1 or T2a b) Intermediate Risk: Any or all of PSA 10–20 ng/mL, Gleason Grade 7 OR Stage T2b-c c) Absence of high risk features (PSA > 20, T3-4, N1 or M1 disease, Gleason score 8–10) with PSA measured within 3 months prior to enrolment, ECOG Performance status 0–2, suitable for definitive external beam radiotherapy (IMRT or VMAT), ability to have three gold fiducial markers placed in the prostate.

#### Exclusion

Prior lymph node irradiation, any other systemic anti-prostate cancer therapy both proven in the metastatic setting and investigational (e.g. docetaxel, enzalutamide, note androgen deprivation therapy is allowed), prostate volume > 90 cm^3^ measured from the CT scan, patient lateral dimension >40 cm as measured at the level of the prostate from the CT scan, two fiducial markers placed closer than 1 cm as measured in the axial CT scan, fiducial migration or fewer than 3 fiducials present in the CT scan.

### Objectives

The primary objective is to quantify accumulated patient dose distributions with the KIM intervention compared to dose distributions estimated without the KIM intervention. Secondary objectives are to assess patient treatment outcomes, to perform a technology assessment of KIM to quantify the clinical practice impact, and to assess KIM cancer targeting accuracy.

### Assessments

For each visit, prostate-specific antigen (PSA), GU and GI physician-graded toxicity (RTOG scale) and patient-reported outcomes using the Expanded Prostate Cancer Index Composite (EPIC) instrument will be recorded. Where possible, and noting the limitations of retrospective comparisons, different patient cohorts and differing eligibility criteria, outcome measures will be compared with controls in participating centres and with large published series, such as the 1100-patient pooled analysis by King [2, 3] and the 477-SBRT patient series from Katz [24].

Physician-reported acute toxicity will be measured during treatment, then after treatment completion at 2 weeks, 6 weeks, 3 months, 6 months and every 6 months thereafter until 3 years after treatment. Patient reported outcomes will be at baseline, then after treatment completion at 2 weeks, 6 weeks, year 1 and year 3. Biochemical control will be assessed with PSA testing at baseline, then after treatment completion at 6 weeks, 3 months, 6 months and every 6 months thereafter until 3 years after treatment. Biochemical failure is defined using the RTOG Phoenix definition [25] (any rise in the PSA >2 ng/L above the nadir). The estimated outcome improvements will be determined by applying established dose-response models to the patient’s accumulated dose and comparing with the estimated accumulated dose distribution in the absence of the KIM intervention.

### Treatment planning

Fiducial markers will be inserted prior to treatment planning. A planning CT is necessary for all patients. MRI is strongly encouraged but not required. Table 1 shows the guidelines for the structures needing to be contoured on the patient’s CT anatomy.

**Table 1:** SPARK organs at risk contouring guidelines

Structure name	Description
Rectum	Contour as a solid structure from recto-sigmoid junction to lower aspect of ischial tuberosities, the latter of which usually corresponds to the anorectal junction.
Bladder	Contour the whole organ as a solid structure. If CT and MR volumes disagree, use the imaging with the smaller bladder volume.
Penile bulb	Contour from MRI, if available, otherwise use CT dataset.
Prostatic urethra planning target at risk volume (PRV)	Estimate urethral position, and add 3 mm radial expansion. If IDC in situ, add 1 mm radial margin to IDC. Please note that a dedicated urethra structure (from which the Urethra_PRV is created) is desirable but not mandatory.
Neck of Femur	Contour the Left and Right NOF as solid structures to the level of the ischial tuberosity.
Remaining volume at risk (RVR)	Defined as the imaged volume within the patient, excluding any delineated OAR and the PTV. The RVR is used to identify unsuspected regions of high absorbed dose (ICRU 83).

The dose-volume constraints for the treatment planning are given in Table 2.

**Table 2:** The treatment planning dose-volume constraints for the SPARK protocol

Constraint	Per-Protocol	Minor Variation	Major Variation
PTV D95%	36.0–36.5Gy (100%)		<36.0Gy or >36.5 Gy
PTV D98%	≥34.44 Gy (95%)	32.72– <34.44 Gy	<32.72 Gy
PTV D2%	≤38.06 Gy (105%)	>38.06–39.96 Gy	>39.96 Gy
PTV Dmax to 0.1 cc	≤38.78 Gy (107%)	>38.78–40.72 Gy	>40.72 Gy
PTV Dmax	Not within a critical structure	N/A	Within a critical structure
Rectum Dmax to 0.1 cc	≤38.06 Gy (105%)	>38.06–39.96 Gy	>39.96 Gy
Rectum V34.4 Gy	≤3 cc	>3–4 cc	>4 cc
Rectum V18.13 Gy	≤50%	>50–60%	>60%
Rectum V29 Gy	≤20%	>20–25%	>25%
Rectum V32.63 Gy	≤5%	>5–10%	>10%
Bladder Dmax to 0.1 cc	≤38.06 Gy (105%)	>38.06 or 39.96 Gy	>39.96 Gy
Bladder V34.4 Gy	≤10 cc	>10–12 cc	>12 cc
Bladder V18.13 Gy	≤50%	>50–60%	>60%
Bladder V32.63 Gy	≤10%	>10–15%	>15%
Urethra_PRV Dmax 0.1 cc	≤38.78 Gy (107%)	>38.78–40.72 Gy	>40.72 Gy
Urethra_PRV V38.0 Gy	≤5%	>5–7%	>7%
FemHead_R, FemHead_L V20 Gy	≤10 cc	>10–14 cc	>14 cc
FemHead_R, FemHead_L Dmax to 0.1 cc	≤30 Gy	>30–32 Gy	>32 Gy
PenileBulb Dmax 0.1 cc	≤36.25 Gy	n/a	n/a
PenileBulb V20 Gy	≤ 1 cc	n/a	n/a
Intermediate dose spillage: ratio of volumes receiving 50% TD to 100% TD	≤4	>4–5	>5
Conformity Index (volume receiving 36.25 Gy/volume of PTV):	≤1.1	>1.1–1.2	>1.2
RVR V36.25 Gy	≤5 cc	>5–7 cc	>7 cc

During treatment planning, the prostate volume will be assessed, patient width will be measured, and the placement, number and inter-marker distance of the fiducials will be assessed. IMRT or VMAT planning is required and both flattened and flattening filer free beams are allowed. 95% of the PTV will be treated to the prescribed dose over the course of 5 fractions. Patients will receive 7.25 Gy per fraction in 5 treatments, consisting of 1–3 fractions per week. Treatment should not be delivered on any 2 consecutive days. Treatment should be completed over a period of no more than 5 weeks. There should be a minimum of 40 h and a maximum of 8 days between fractions.

### Treatment delivery

Prior to the IMRT or VMAT delivery, a Cone Beam Computed Tomography (CBCT) scan will be acquired. The patient will be aligned to the planned treatment position based on the CBCT. KIM will be initiated just prior to treatment and will monitor the prostate position as the treatment beam is delivered. Two methods will be allowed to manage movement during treatment, couch shift and multileaf (MLC) tracking. If a couch shift adaptive strategy is used, when the prostate moves from its planned position by more than 2 mm for more than 5 s, the treatment will be interrupted and the patient realigned based on the KIM software so that the prostate target is aligned to its planned position. Lower action thresholds and more frequent corrections are allowed at the discretion of the treatment team. If an MLC tracking adaptive strategy is used, the beam will continually be adjusted to target the prostate tumour.

### Sample size calculation

We will enrol 48 patients to test the hypothesis that patient dose distributions are improved with the use of the KIM technology. The derivation follow: treating each treatment session as an independent event and then using Simon’s two-stage design, a sample size of 24 sessions with intervention events will give us 90% power with 95% confidence to rule in a success rate of 2/3 in favour of the futile rate of 1/3. To obtain an estimated 24 treatment sessions with intervention events we need 24/0.10 = 240 treatment sessions, which for the 5-session SBRT regime equals 48 patients. The null hypothesis will be rejected if 16 or more responses are observed in 48 patients. This design yields a type I error rate of 0.0488 and power of 0.91 when the true response rate is 67%.

### Analysis

An interim analysis will be performed after 20 patients have been accrued. This will correspond to an estimated 10 intervention events over 100 sessions. If four or fewer responses are observed in these 10 events, consideration will be given to the cause of the lack of response and the study may be stopped on the grounds of futility. At the same time, the IDSMC will review the trial for safety and determine whether or not it should continue. Otherwise, the study will continue with an additional 28 patients to be recruited. The main analysis will be performed after all 48 patients have completed treatment, with the final analysis being performed after the last patient has completed 2 years of follow up.

## Discussion

The clinical trial **SPARK**: **S**tereotactic **P**rostate **A**daptive **R**adiotherapy utilizing **K**ilovoltage Intrafraction Monitoring (KIM) is an example of bench-to-bedside research translation into a phase II trial. KIM is a real-time image guided radiotherapy technology being clinically pioneered in targeted prostate cancer radiotherapy. It has potential for widespread use for cancers of the pelvis, thorax and abdomen. Real-time radiotherapy has several benefits for patients. In addition to the increased geometric accuracy, leading to improve dosimetric target and normal tissue coverage – and the expected commensurate improvement in clinical outcomes – real-time radiotherapy has the potential to improve the throughput of cancer treatments and reduce the imaging dose required.

The improvement in throughput comes as the real-time imaging can replace the time consuming pre-treatment and intra-treatment volumetric imaging procedures, such as cone beam CT, as well as the time taken to analyse these images. With the imaging being acquired during the treatment, and the analysis being automated, there is potential for improved throughput.

The reduction in the imaging dose is due to the elimination in the use of repeat cone beam CT scans before treatment, and the elimination of the use of cone beam CT scans between treatments. Though KIM does use the x-ray imager during treatment, the reduced field size – 6 × 6 cm^2^ compared to ≥25 × 25 cm^2^ with cone beam CT for each projection – means a much lower imaging dose, even if the number of projections is higher and/or the dose per image is higher.

## Abbreviations

ASTRO: American Society for Radiation Oncology
IDSMC: Independent Data Safety Monitoring Committee
IMRT: Intensity Modulated Radio Therapy
KIM: Kilovoltage Intrafraction Monitoring
MLC: Multileaf Collimator
PSA: Prostate-Specific Antigen
SBRT: Stereotactic Body Radiation Therapy
SPARK: Stereotactic Prostate Adaptive Radiotherapy utilising Kilovoltage Intrafraction Monitoring
TROG: Trans-Tasman Radiation Oncology Group (TROG Cancer Research)
VMAT: Volumetric Modulated Arc Therapy

## References

[1] Pan H, Simpson DR, Mell LK, Mundt AJ, Lawson JD (2011). A survey of stereotactic body radiotherapy use in the United States. Cancer.

[2] King CR, Collins S, Fuller D, Wang P-C, Kupelian P, Steinberg M, Katz A (2013). Health-Related Quality of Life After Stereotactic Body Radiation Therapy for Localized Prostate Cancer: Results From a Multi-institutional Consortium of Prospective Trials. Int J Radiat Oncol Biol Phys.

[3] King CR, Freeman D, Kaplan I, Fuller D, Bolzicco G, Collins S, Meier R, Wang J, Kupelian P, Steinberg M, Katz A (2013). Stereotactic body radiotherapy for localized prostate cancer: Pooled analysis from a multi-institutional consortium of prospective phase II trials. Radiother Oncol.

[4] ASTRO Model Policy, Stereotactic Body Radiation Therapy. ASTRO web site 2013.

[5] Zelefsky MJ, Kollmeier M, Cox B, Fidaleo A, Sperling D, Pei X, Carver B, Coleman J, Lovelock M, Hunt M (2012). Improved clinical outcomes with high-dose image guided radiotherapy compared with non-IGRT for the treatment of clinically localized prostate cancer. Int J Radiat Oncol Biol Phys.

[6] Singh J, Greer PB, White MA, Parker J, Patterson J, Tang CI, Capp A, Wratten C, Denham JW (2013). Treatment-Related Morbidity in Prostate Cancer: A Comparison of 3-Dimensional Conformal Radiation Therapy With and Without Image Guidance Using Implanted Fiducial Markers. Int J Radiat Oncol Biol Phys.

[7] Gill S, Thomas J, Fox C, Kron T, Rolfo A, Leahy M, Chander S, Williams S, Tai KH, Duchesne G, Foroudi F (2011). Acute toxicity in prostate cancer patients treated with and without image-guided radiotherapy. Radiat Oncol.

[8] Ratnayake G, Martin J, Plank A, Wong W. Incremental changes verses a technological quantum leap: The additional value of intensity-modulated radiotherapy beyond image-guided radiotherapy for prostate irradiation. J Med Imaging Radiat Oncol. 2014;n/a-n/a 25243269

[9] Report of the ASTRO Emerging Technology Committee. Stereotactic Body Radiotherapy (SBRT) For Primary Management of Early-Stage, Low-Intermediate Risk Prostate Cancer. 2008.

[10] King CR, Brooks JD, Gill H, Pawlicki T, Cotrutz C, Presti JC (2009). Stereotactic body radiotherapy for localized prostate cancer: interim results of a prospective phase II clinical trial. Int J Radiat Oncol Biol Phys.

[11] Kupelian P, Willoughby T, Mahadevan A, Djemil T, Weinstein G, Jani S, Enke C, Solberg T, Flores N, Liu D, Beyer D, Levine L (2007). Multi-institutional clinical experience with the Calypso System in localization and continuous, real-time monitoring of the prostate gland during external radiotherapy. Int J Rad Onc Biol Phys.

[12] Sandler HM, Liu P-Y, Dunn RL, Khan DC, Tropper SE, Sanda MG, Mantz CA (2010). Reduction in Patient-reported Acute Morbidity in Prostate Cancer Patients Treated With 81-Gy Intensity-modulated Radiotherapy Using Reduced Planning Target Volume Margins and Electromagnetic Tracking: Assessing the Impact of Margin Reduction Study. Urology.

[13] Lindencrona U, Braide K, Syrén H, Hertzman S, Kindblom J (2012). Clinical Experience of Real-Time Tracking with the Raypilot System in Patients with Prostate Cancer (ESTRO Poster). Radiother Oncol.

[14] Kitamura K, Shirato H, Seppenwoolde Y, Onimaru R, Oda M, Fujita K, Shimizu S, Shinohara N, Harabayashi T, Miyasaka K (2002). Three-dimensional intrafractional movement of prostate measured during real-time tumor-tracking radiotherapy in supine and prone treatment positions. Int J Radiat Oncol Biol Phys.

[15] Poulsen PR, Cho B, Keall PJ (2009). Real-time prostate trajectory estimation with a single imager in arc radiotherapy: a simulation study. Phys Med Biol.

[16] Poulsen PR, Cho B, Langen K, Kupelian P, Keall PJ (2008). Three-dimensional prostate position estimation with a single x-ray imager utilizing the spatial probability density. Phys Med Biol.

[17] Poulsen PR, Cho B, Sawant A, Keall PJ (2010). Implementation of a new method for dynamic multileaf collimator tracking of prostate motion in arc radiotherapy using a single kV imager. Int J Radiat Oncol Biol Phys.

[18] Poulsen PR, Cho B, Sawant A, Ruan D, Keall PJ (2010). Dynamic MLC tracking of moving targets with a single kV imager for 3D conformal and IMRT treatments. Acta Oncol.

[19] Ng J, Booth J, O’Brien R, Colvill E, Huang C-Y, Poulsen PR, Keall P (2014). Quality assurance for the clinical implementation of kilovoltage intrafraction monitoring for prostate cancer VMAT. Med Phys.

[20] Ng JA, Booth JT, Poulsen PR, Fledelius W, Worm ES, Eade T, Hegi F, Kneebone A, Kuncic Z, Keall PJ (2012). Kilovoltage intrafraction monitoring for prostate intensity modulated arc therapy: first clinical results. Int J Radiat Oncol Biol Phys.

[21] Keall PJ, Ng JA, Juneja P, O’Brien RT, Huang C-Y, Colvill E, Caillet V, Simpson E, Poulsen PR, Kneebone A (2016). Real-Time 3D Image Guidance Using a Standard LINAC: Measured Motion, Accuracy, and Precision of the First Prospective Clinical Trial of Kilovoltage Intrafraction Monitoring–Guided Gating for Prostate Cancer Radiation Therapy. Int J Radiat Oncol Biol Phys.

[22] Keall PJ, Ng JA, O’Brien R, Colvill E, Huang C-Y, Poulsen PR, Fledelius W, Juneja P, Simpson E, Bell L (2015). The first clinical treatment with kilovoltage intrafraction monitoring (KIM): A real-time image guidance method. Med Phys.

[23] National Comprehensive Cancer Network (2016). NCCN Clinical Practice Guidelines in Oncology: Prostate Cancer.

[24] Katz AJ, Kang J (2014). Stereotactic body radiotherapy as treatment for organ confined low-and intermediate-risk prostate carcinoma, a 7-year study. Front Oncol.

[25] Roach M, Hanks G, Thames H, Schellhammer P, Shipley WU, Sokol GH, Sandler H (2006). Defining biochemical failure following radiotherapy with or without hormonal therapy in men with clinically localized prostate cancer: Recommendations of the RTOG-ASTRO Phoenix Consensus Conference. Int J Radiat Oncol Biol Phys.

